# Centers for Oceans and Human Health: contributions to an emerging discipline

**DOI:** 10.1186/1476-069X-7-S2-S1

**Published:** 2008-11-07

**Authors:** Edward A Laws, Lora E Fleming, John J Stegeman

**Affiliations:** 1 School of the Coast and Environment, 1002 K Energy, Coast and Environment Building, Louisiana State University, Baton Rouge, Louisiana 70803-4110, USA; 2 Departments of Epidemiology & Public Health and Marine Biology & Fisheries, Miller School of Medicine and Rosenstiel School of Marine and Atmospheric Sciences, University of Miami, Clinical Research Building, 10th Floor (R669), 1120 NW 14th Street, Miami, Florida, USA; 3 Woods Hole Oceanographic Institution, Woods Hole Center for Oceans and Human Health, Woods Hole, MA 02543, USA

## 

The oceans are the dominant feature of the planet and are fundamentally linked to human history and to human health. Concerns about the impact of the oceans on human health can be traced to ancient times. Jewish law prohibited the consumption of shellfish, probably reflecting the fact that filter-feeding bivalves can accumulate pathogens and toxins. The Portuguese explorer Pedro Fernandes de Queirós described symptoms associated with ciguatera fish poisoning after eating Caribbean sea bream in 1606, and several of British explorer James Cook's crew experienced similar symptoms after eating fish off the coast of Vanuatu in 1774 [1]. Roughly 1,200 people died from the consumption of fish and shellfish contaminated with methyl mercury in Minamata (Japan) during the 20^th ^century; an even larger number were affected by chronic long-term neurotoxicological impacts [2]. A tsunami caused by an undersea earthquake on December 26, 2004 killed more than 225,000 people in eleven countries bordering the Indian Ocean; and more than 1,400 people died within a single day when the storm surge generated by Hurricane Katrina overwhelmed the New Orleans levee system on August 29, 2005 [3]. Looking ahead, the International Panel on Climate Change has projected a sea level rise of as much as 88 cm during the 21^st ^century as a result of global warming [4], with major implications for the welfare and sustainability of coastal communities.

Despite concerns about biological, physical and chemical risks, and the possible long-term effects of global warming, the oceans remain a resource of immense value to mankind. Many marine fish and shellfish, for example, are excellent sources of protein, selenium, and omega-3 polyunsaturated fatty acids. Around the globe, the coastal ocean is a recreational destination for a substantial percentage of the human population as well as a venue of intensive commercial activity. In 1999, the National Research Council (NRC) published From Monsoons to Microbes: Understanding the Oceans Role in Human Health [5], a seminal document that examined both positive and negative implications of ocean phenomena for human health. The NRC report catalyzed a series of reports [6-11] and activities related to the impacts of the ocean on human health and human impacts on oceans, and suggested how efforts could be directed to anticipate and respond to health needs and threats.

Recognizing the growing need for interdisciplinary basic and applied research addressing the linkages between human health and the oceans, in 2004 the National Science Foundation (NSF) and the National Institute of Environmental Health Science (NIEHS) jointly funded grants establishing four Centers for Oceans and Human Health (COHH). These centers are: (i) the Oceans and Human Health Center at the University of Miami, (ii) the Pacific Research Center for Marine Biomedicine at the University of Hawaii, (iii) the Pacific Northwest Center for Human Health and Ocean Studies at the University of Washington, and (iv) the Woods Hole Center for Oceans and Human Health at the Woods Hole Oceanographic Institution, the Marine Biological Laboratory, and Massachusetts Institute of Technology. Through an internal competitive process, the National Oceanic and Atmospheric Administration (NOAA) in the same year designated three NOAA centers of excellence in oceans and human health under their Oceans and Human Health Initiative (OHHI): (i) the West Coast Center for Oceans and Human Health at the Northwest Fisheries Science Center in Seattle, Washington, (ii) the NOAA Center of Excellence in Oceans and Human Health at the Hollings Marine Laboratory in Charleston, South Carolina, and (iii) the NOAA Center of Excellence for Great Lakes and Human Health at the Great Lakes Environmental Research Laboratory in Ann Arbor, Michigan. The NOAA initiative also includes external competitive grant, distinguished scholar, and traineeship programs.

In the United States, one of the motivations for OHH research arises from the fact that some 62 million Americans swim in the coastal waters of the United States and spend 800 million person-days at beaches annually [12]. Wound, eye and ear infections that can result from pathogen exposure often go unreported, but may be serious; for example, *V. vulnificus *wound infections have a death rate of some 50% [13]. Public awareness of the healthful characteristics of many seafoods is reflected by increasing per capita seafood consumption by U.S. citizens to more than 7 kg per year in 2003. However, the presence of algal toxins and chemical pollutants in some seafood products, and exposure to microbial contaminants (such as *Vibrio *bacteria and *Noroviruses*) as a result of the consumption of raw and even cooked molluscan shellfish remain a concern [14,15]. The economic costs associated with the impact of harmful algal blooms (HABs) on public health have been estimated to be at least $20 million per year in the United States; while in Southern California alone, the medical costs associated with microbial contamination of recreational beaches range between $20 million and $50 million per year [12].

Oceans and Human Health is a growing integration of several disciplines, from physical oceanography to molecular biology to epidemiology, emerging largely from the recognition that ocean processes have important implications for public health, and human activities can influence these processes. Details on approaches to this new area of concern may be found in the Interagency Oceans and Human Health Research Implementation Plan [16]. The five articles in this Oceans and Human Health Supplement issue of Environmental Health were conceived at a joint meeting of investigators from the NSF/NIEHS and the NOAA Oceans and Human Health Centers. Their intent is to articulate some of the challenges that lie ahead in this new multi-disciplinary area of scientific research [17]. The papers focus in part on prevention, particularly for HABs and pathogens, since one strategy in Oceans and Human Health is to "get ahead of the curve", i.e., to warn the public and resource managers before morbidity and mortality rates start to rise.

## Microbial pathogens and OHH

The primary concern regarding microbial pathogens is human exposure associated with the recreational and commercial use of coastal waters containing either indigenous or introduced pathogens. The primary route of transmission is the consumption of seafood, but can occur also by contact with seawater or exposure to marine aerosols and zoonoses. Almost all major bacterial lineages that contain human pathogens have also been detected in samples from ocean environments or organisms[18]. Yet for only few of these pathogens are tests available, and even fewer are routinely monitored [18].

For years, the standard strategy for monitoring introduced pathogens has been measuring the concentration of so-called fecal indicator bacteria (FIB). There are acknowledged and serious problems associated with the use of FIB for this purpose [19]. These include the facts that: (i) there is large spatial and temporal variability in FIB concentrations at recreational beaches, particularly in sub/tropical areas; (ii) FIB are not uniquely diagnostic for human fecal pollution; (iii) FIB may or may not survive in the environment as long as some of the hardier fecal pathogens (e.g., infectious hepatitis) for which they are the alleged surrogates; (iv) the standard assays for FIB take 24–48 hours; (v) only some strains of pathogenic microorganisms are highly virulent; and (vi) FIB provide no indication of the presence or abundance of non-enteric pathogens (e.g., *Staphyllococcus aureus*, *Naegleri fowleri *and *Legionella pneumophila*). Recent epidemiological studies conducted at recreational beaches impacted by non-point source pollution have revealed no statistically significant correlation between FIB concentrations and morbidity [19]. These issues are being addressed through a multidisciplinary and innovative strategy, including: (i) tests for microbes truly diagnostic of human and animal fecal pollution; (ii) molecular assays that are far more rapid than culture techniques; (iii) tests for the presence of virulence genes; (iv) the use of sentinel species and habitats [12,19]; and (v) the use of mathematical models to fill in spatial and temporal gaps in the data, and predict the behavior of complex systems [20].

Polymerase chain reaction methods for example, are being used to rapidly and specifically target microbes of public health concern, including those not examined previously because of an inability to culture them. New molecular assays have been introduced for detection of FIB, bacterial pathogens, viral pathogens, and protistan (protozoan) parasites. And recent methodological improvements are allowing simultaneous detection of multiple targets in a single assay. Scientists from MIT in the Woods Hole Center, in collaboration with colleagues from Northwestern University, have recently developed a protocol involving DNA microarrays that enables accurate identification of specific bacterial sequences in natural samples. Applied to environmental samples, the microarrays are able to detect *Vibrio cholerae *at natural concentrations in New England estuarine water, suggesting that microarrays may be applicable to broad environmental monitoring of co-occurring pathogens [19]. OHH researchers are working with the Southern California Coastal Water Research Project and the U. S. Environmental Protection Agency to evaluate the potential for such novel microbial detection technologies to forecast human health risks at California beaches contaminated with non-point sources of pollution. Researchers at the University of Miami are currently conducting an epidemiological study at Florida beaches impacted by non-point source pollution in collaboration with the U.S. Centers for Disease Control and Prevention and Florida Dept of Health [19].

## Harmful algal blooms (HAB) and OHH

The acronym "HAB" is somewhat misleading since some HAB-designated problems (e.g., ciguatera fish poisoning), though caused by algae, are not associated with blooms in any conventional sense of the word, and others are caused by cyanobacteria, which are prokaryotic and, strictly speaking, not algae. Regardless, there are a variety of human health problems associated with HABs. The most common is acute (and in some cases, chronic) intoxication resulting from the consumption of shellfish or finfish that contain neurotoxins produced by HABs [21]. Other problems include respiratory irritation caused by the inhalation of aerosolized toxins produced by HABs, and the presence of HAB toxins in drinking water supplies, as in the Great Lakes.

In some cases remote sensing has been very successful in detecting HABs (and warning the public). A good example is detection of *Karenia brevis *blooms off the west coast of Florida by scientists at the University of Miami COHH in collaboration with NOAA OHH researchers [21]. Scientists at the University of Washington COHH have developed a prototype antibody-based sensor for the detection of domoic acid, the neurotoxin that causes amnesic shellfish poisoning, and are collaborating with the University of Hawaii COHH and the Hollings Marine Laboratory to develop a similar probe for ciguatoxin [21]. In Puget Sound, the toxicity of shellfish is being monitored as a proxy to detect HAB events, as monitoring of phytoplankton abundance and composition is not sufficiently frequent [22]. As with pathogens, mathematical models have been used to integrate information from field studies and, in combination with biological mechanisms, to gain insight into the processes that trigger algal blooms. One of the best examples of this approach has been the hindcasting of *Alexandrium fundyense *blooms in the Gulf of Maine by the Woods Hole COHH [20,21,23].

A concern with modeling and forecasting is the growing awareness that the abundance and, in particular, the toxicity of HAB species may not be explainable solely in terms of the physics and chemistry of the environment. Interactions with bacteria and/or viruses, for example, may play an important role in toxin production. Interactions with bacteria appear to play a role in the production of the neurotoxin, domoic acid, by the diatom, *Pseudo-nitzschia *[21]. The importance of such species interactions has been established also among pathogens; *Vibrio cholerae*, for example acquires the cholera toxin gene via phage transduction [19],

Another emerging issue has been the discovery by scientists at the Hawaii COHH and others that the neurotoxin, β-N-methylamino-L-alanine (BMAA), is produced by all known groups of cyanobacteria [21]. BMAA has been suggested to contribute to neurodegenerative diseases such as amylotrophic lateral sclerosis (ALS)/Parkinson's Disease and Alzheimer's-dementia and similar diseases in humans and animals [24]. It may find its way into the diets of humans and wildlife through the consumption of cyanobacteria, food chain magnification, and/or exposure to contaminated water supplies [21]. Currently, our concern about the impact of most HAB toxins on human health, and consequently our ability to mitigate the risks, is based largely on risks from acute exposures. However, there is growing concern about potential risks associated with long-term exposure to BMAA or HAB-associated toxins. Consumption of drinking water and/or seafood, for example, might result in chronic exposure to low levels of toxins, at concentrations below regulatory levels or even conventional detection limits [21].

## Global change and OHH

In terms of human health effects worldwide, a very important yet poorly understood issue emerging in OHH is the potential impact of climate change on the ecology of pathogens and harmful algal species. Among the eukaryotic microalgae, dinoflagellates are expected to benefit from a warmer climate and more thermally stratified water column [22]. Most HAB species are dinoflagellates. Global warming is expected to accelerate hydrological cycles, with resultant increases in stream runoff to coastal waters surrounding much of Asia and North and South America. Nutrient loading associated with increased coastal runoff may trigger more frequent blooms of HA species in the oceans and in fresh waters [22]. One of the proposed strategies for mitigating the impact of anthropogenic CO_2 _emissions, fertilization of large areas of the ocean with iron, could also stimulate HABs. Field studies of the impact of iron fertilization by researchers at the Hawaii OHH Center have indicated that pennate diatoms are the primary beneficiaries of iron fertilization; *Pseudo-nitzschia*, the genus containing a number of species associated with the production of the neurotoxin domoic acid and amnesiac shellfish poisoning, is particularly responsive [22].

## Outreach and education

Various agencies, in part through the Centers, are helping to train a new generation of scientists in the interdisciplinary aspects of OHH through the participation of students and post-docs in OHH research projects and related activities, including the NSF-funded OHH research experience for undergraduates (REU) program and a new NOAA-funded OHH Traineeship Program.

Collaborations among the various OHH centers and other OHH researchers have: (i) produced a noteworthy multi-center and interdisciplinary microbiological study of New Orleans and Lake Pontchartrain following Hurricane Katrina [25]; (ii) produced a special edition of Oceanography on OHH [26] and a textbook on OHH [27]; and (iii) helped facilitate the organization of multiple sessions on OHH at the 2006 and 2008 AGU/ASLO/TOS Ocean Sciences meetings, as well as a variety of other symposia, workshops, and meetings and a new Gordon Research Conference-supported conference and Graduate Research Seminar on Oceans and Human Health . Synergistic and collaborative contributions to the ongoing work of the Interagency Working Group on Harmful Algal Blooms, Hypoxia and Human Health have played an important role in the success of the Working Group in producing the first comprehensive assessment of the status of OHH research and opportunities for advancement in this new field [17]. Hopefully, the current volume will provide a perspective on the current status in several areas, which may stimulate further efforts in this young and globally important endeavor and scientific discipline.

## Competing interests

The authors declare that they have no competing interests.
